# A CMOS-Thyristor Based Temperature Sensor with +0.37 °C/−0.32 °C Inaccuracy

**DOI:** 10.3390/mi11020124

**Published:** 2020-01-22

**Authors:** Jing Li, Yuyu Lin, Siyuan Ye, Kejun Wu, Ning Ning, Qi Yu

**Affiliations:** University of Electronic Science and Technology of China, Chengdu 610054, China

**Keywords:** temperature sensor, CMOS thyristor, VCO

## Abstract

This paper describes a voltage controlled oscillator (VCO) based temperature sensor. The VCOs are composed of complementary metal–oxide–semiconductor (CMOS) thyristor with the advantage of low power consumption. The period of the VCO is temperature dependent and is function of the transistors’ threshold voltage and bias current. To obtain linear temperature characteristics, this paper constructed the period ratio between two different-type VCOs. The period ratio is independent of the temperature characteristics from current source, which makes the bias current generator simplified. The temperature sensor was designed in 130 nm CMOS process and it occupies an active area of 0.06 mm^2^. Based on the post-layout simulation results, after a first-order fit, the sensor achieves an inaccuracy of +0.37/−0.32 °C from 0 °C to 80 °C, while the average power consumption of the sensor at room temperature is 156 nW.

## 1. Introduction

Temperature sensors are desirable for temperature sensing and compensation in modern applications, such as medical, environmental monitoring, thermal monitoring and wireless Internet-of-Things (IoT) platforms [1,2,3]. Most of such applications are battery powered for portability, durability or deployment flexibility [4,5], and put a strict power budget on temperature sensor which drives the power consumption to sub-μW and even to near-zero.

Prior works have demonstrated low-power-consumption temperature sensors based on bipolar junction transistors (BJT) [6,7,8], resistors [9,10], and complementary metal–oxide–semiconductor (CMOS) [11,12,13,14,15,16]. BJT-based temperature sensors converse the temperature by comparing a temperature-dependent voltage to a temperature-insensitive voltage or inversely temperature-dependent voltage. The voltage difference between these two voltages is quantized by a ΣΔ-ADC (Analog-to-Digital Converter), which achieves high resolution up to 0.002 °C and inaccuracy less than ±0.2 °C [6,7,8]. Resistor-based temperature sensors can achieve higher resolution and energy efficiency than BJT-based sensors. As discussed in Ref. [9], the authors use a temperature-dependent RC (Resistor and Capacitor) filter to extract the temperature information. In Ref. [10], the authors build a resistor based Wheatstone bridge whose voltage difference is proportional to the temperature. Both the temperature-dependent phase shift and voltage difference are sequentially digitized by ΣΔ-ADCs. ΣΔ-ADCs achieve high resolution at the cost of high power consumption and hardware cost from the decimation filter which normally are not mentioned in papers. Thus, the sacrifice of power consumption and hardware make BJT and resistor based temperature sensors not an optimal choice for battery powered applications. CMOS based temperature sensors are normally composed of digital delay cells. They are much more area efficient and easier to scale down with process. The authors in Ref. [13] demonstrate a VCO based temperature sensor. The temperature information is extracted by two VCOs with different temperature-dependent frequency. The temperature sensor is fully composed of digital circuit and thus achieves an attractive area. On the other hand, the authors in Ref. [12] firstly create a temperature-dependent current and then transfer the current to frequency by VCOs. It provides better linearity and accuracy.

This paper proposes a VCO based temperature sensor and the VCO is composed of two-stage CMOS thyristor to simplify the circuit and achieve low power consumption.

## 2. Proposed CMOS Thyristor Based VCO

The proposed VCO is composed of multi-stage delay cells circled together. The desired oscillation frequency is inversely proportional to the number of stages and delay time of delay cell. Increasing the stages or delay time is necessary to reduce the oscillation frequency for low power consumption. Commonly, CMOS invertor is adopted as delay cell for its simplicity and scalability [12,13,14,15,16]. Its delay time is process dependent and ranges from ps to ns in sub-µm CMOS process. It may achieve µs delay by using the current-starved architecture but at the cost of large capacitors [14,15,16].

### 2.1. Basics of CMOS Thyristor Based Delay Cell

**Figure 1:**
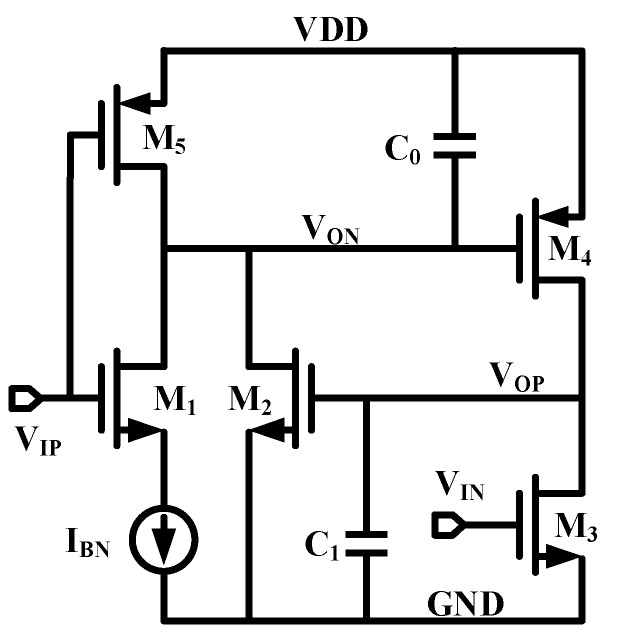
Complementary metal–oxide–semiconductor (CMOS) thyrsitor based delay cell.

CMOS thyristor was proposed to increase the delay [17,18]. As shown in Figure 1, M_2_ and M_4_ work as a thyristor. M_3_ and M_5_ receive the input signal *V_IP_* and *V_IN_*, respectively, while *V_IN_* is the complementary signal of *V_IP_*. Current source *I_BN_* is used to sink current from *V_ON_* via switch M_1_ according to the status of *V_IP_*. C_0_ is the total capacitance on node *V_ON_*. When *V_IP_* is low, M_5_/M_3_ are conducted and M_1_ is non-conducted, *V_ON_* and *V_OP_* are reset to *VDD* and *GND*, respectively. When *V_IP_* is changing to high, M_5_/M_3_ switch off and M_1_ switches on. Current source *I_BN_* starts sinking current from *V_ON_* which results in node voltage decreasing from *VDD*. Approximately, M_4_ conducts when *V_IP_* reaches *VDD*-*V_thp_*, where *V_thp_* is the threshold voltage of M_4_. After that, *V_OP_* is charged from power supply *VDD* via M_4_ and increased rapidly. Sequentially, M_2_ conducts when *V_OP_* reaches *V_thn_* and discharges current from *V_ON_* together with *I_BN_*, where *V_thn_* is the threshold voltage of M_2_. It accelerates the node voltage decrease on *V_ON_* and constitutes a positive feedback loop between M_2_ and M_4_. Once the positive feedback is built, *V_ON_* and *V_OP_* toggle to the opposite state in a short time. The corresponding delay value is calculated as [17]

(1) $$td=\frac{C_{0}V_{thp}}{I_{BN}}+\sqrt{\frac{6C_{1}{C_{0}}^{2}}{\kappa {I_{BN}}^{2}}V_{thn}}+\delta t$$

where the first term on the right is the delay contributed by discharging C_0_, the second term is the delay contributed by charging C_1_, and *δt* is the regeneration time of the CMOS thyristor. C_1_ is the parasitic capacitance of *V_OP_*. In reality, because of the subthreshold effect, M_4_ charges the C_1_ simultaneously with M_2_ discharging C_0_. The second term can be neglected. Comparing to the first term, the regeneration time *δt* is small and can be omitted. The delay value can be approximated as

(2) $$td\approx \frac{C_{0}V_{thp}}{I_{BN}}$$

Both adopting a larger capacitor *C*_0_ and reducing sink current *I_BN_* are effective to increase the delay value. Since *C*_0_ generally adopts metal capacitor which is mostly temperature independent, the temperature coefficients are decided by the ratio of *V_thp_*/*I_BN_*.

### 2.2. Proposed CMOS Thyristor Based VCO

**Figure 2:**
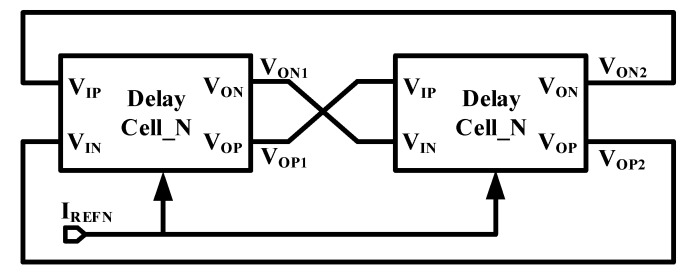
Proposed CMOS thyristor based voltage controlled oscillator (VCO).

VCO is generally constituted by multiple stages (beyond three stages) to satisfy the desired frequency. The more stages it has, the more devices are required, which would consume more power and also deteriorate the wave performance. To simplify the circuit and reduce device counts, a two-stage ring VCO is proposed and shown in Figure 2. The delay cell is composed of the CMOS thyristor based delay cell aforementioned. In the proposed architecture, the negative output *V_ON_*_1_ of the first stage is connected to the negative input *V_IN_* of the second stage and *V_OP_*_1_ is connected to *V_IP_*. In this way, four-stage inversion is produced in the closed loop and thus makes it easy to satisfy the oscillation requirement of 360° phase shift.

Referred to the delay value of CMOS thyristor based delay cell in Equation (1), the oscillation period of the proposed VCO is given by

(3) $$T_{VCO\_P}=\frac{2C_{0}V_{thp}}{I_{BN}}$$

## 3. Temperature Sensor Architecture

**Figure 3:**
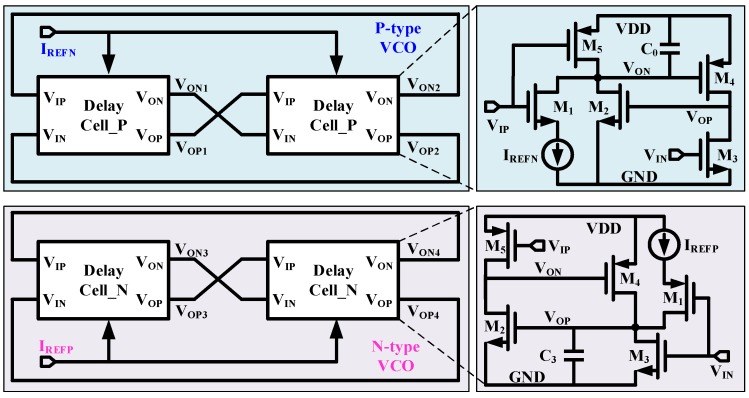
Proposed temperature sensor architecture.

As shown in Figure 3, two types of VCO are adopted to constitute the proposed temperature sensor. At the top, P-type VCO is composed of Delay_Cell_Ps. The delay value of Delay_Cell_P is approximately the discharging time on *V_ON_* via *I_REFN_* according to aforementioned analysis. Referring to Equation (3), the period of P-type VCO is given as

(4) $$T_{VCO\_P}=\frac{2C_{0}V_{thp}}{I_{REFN}}$$

Similarly, N-type VCO is composed of Delay_Cell_Ns. The delay value of Delay_Cell_N is approximately the charging time on *V_OP_* via *I_REFP_* according to aforementioned analysis. Referring to Equation (3), the period of N-type VCO is given as

(5) $$T_{VCO\_N}=\frac{2C_{3}V_{thn}}{I_{REFP}}$$

Based on Equations (4) and (5), the ratio between *T_VCO_P_* and *T_VCO_N_* is calculated

(6) $$ratio\_T=\frac{T_{VCO\_P}}{T_{VCO\_N}}=\frac{C_{0}I_{REFP}}{C_{3}I_{REFN}}\frac{V_{thp}}{V_{thn}}$$

C_0_ and C_3_ are composed of metal capacitors, which are temperature independent. *I_REFP_* and *I_REFN_* come from the same current source and they will have the same temperature characteristics and value. Thus, the ratio can be updated as

(7) $$ratio\_T\propto \frac{V_{thp}}{V_{thn}}$$

Because *V_thp_* is temperature dependent to the first order [19], *ratio_T* is proportional to the temperature [13]. Thus, the ratio of the two-type VCO is used to detect temperature.

**Figure 4:**
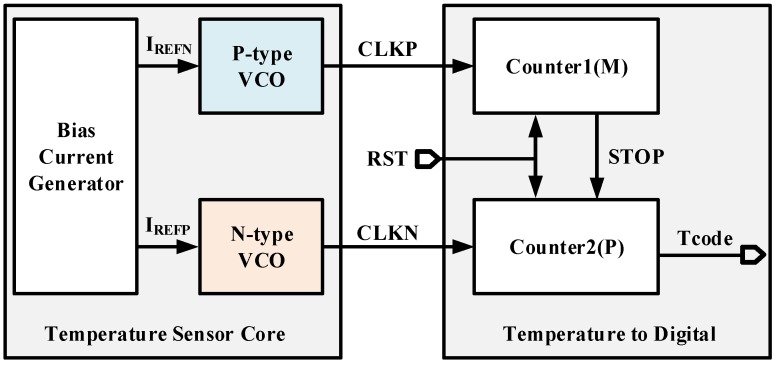
Block diagram of the temperature sensor.

The complete block diagram of the proposed temperature sensor is shown in Figure 4. The left block is temperature sensor core which is composed of a P-type and a N-type VCO aforementioned. Bias current generator is used to provide the charging/discharging current *I_REFN_*/*I_REFP_*.

The right block calculates the ratio between periods and transfers temperature to digital codes via two counters. The working principle of the *Temperature to Digital* is as follows. After *RST* switches to low, both counters start counting simultaneously. Counter1 counts *CLKP* to a constant value *M* and generates a flag signal *STOP*. When receiving *STOP* signal, Counter2 ends counting *CLKN* with a value of *P.* The counting time of both counters are equal and it can be expressed as

(8) $$M\cdot T_{VCO\_P}=P\cdot T_{VCO\_N}$$

The ratio between periods can be obtained

(9) $$ratio\_T=\frac{T_{VCO\_P}}{T_{VCO\_N}}=\frac{P}{M}$$

Since *M* is a constant value, *P* represents the *ratio_T* and thus is proportional to the temperature. Eventually, *P* is converted to digital code of *Tcode* and output for measurement.

## 4. Circuit Implementation

### 4.1. Delay Cell

**Figure 5:**
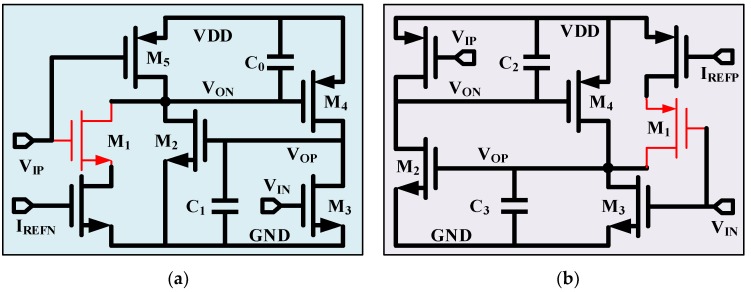
Detail of delay cell (**a**) Delay_Cell_P (**b**) Delay_Cell_N.

The detail of delay cell is shown in Figure 5. The bias current *I_REFP_* and *I_REFN_* are composed of NMOS and PMOS working in saturation region separately. The switches M_1_ in both delay cells adopt thin-gate MOSFET to achieve fast switching and low conductive resistance. Considering the charging/discharging current of *I_REFP_*/*I_REFN_* on the magnitude of nA, all the others are thick-gate MOSFET to reduce influence of leakage current. Instead of just using C_0_ and C_3_ as what is shown in Figure 3, C_1_ and C_2_ are added to keep the load balance between Delay_Cell_P and Delay_Cell_N. MIM (Metal-Insulator-Meatal) capacitors are adopted for C_0_-C_3_ to maintain process and temperature independence.

### 4.2. Bias Current Generator

**Figure 6:**
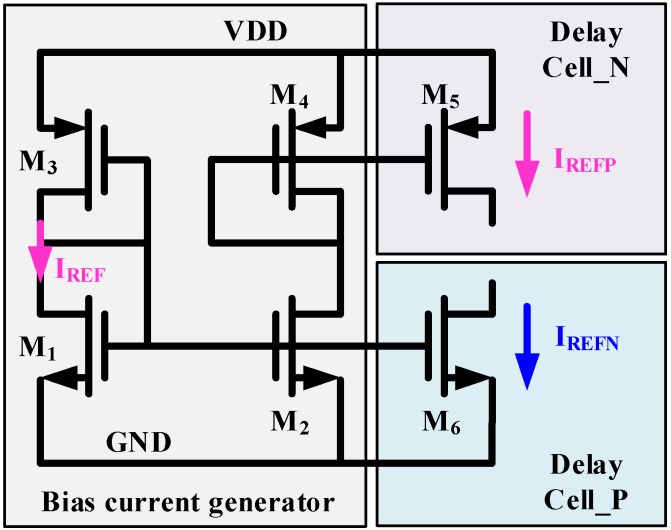
Bias current generator.

Bias current could be designed with any kind of temperature characteristics, such as proportional, exponential, or independent [12,20]. It is hard to obtain a temperature dependent or independent current with outstanding linearity even at the cost of power or hardware. Instead of designing a high-linearity and temperature-dependent current generator, this paper adopted a simple current generator since the proposed temperature sensor is independent of *I_BN_*. Figure 6 shows the architecture of the bias current generator.

As described in Equation (6), *I_REFP_* and *I_REFN_* can be removed as long as they have the same temperature characteristics and magnitude. Thus, the bias current generator is simply composed of M_1_-M_4_. M_1_ and M_3_ work in sub-threshold region and provide the source current *I_ref_*.

(10) Iref=μpμnCoxpCoxnWpLpWnLn(m−1)VT2exp(VDD−Vthn−Vthp2mVT)

where *μ* is mobility, *C_ox_* is oxide capacitance, *W* is transistor width, *L* is transistor length, *m* is subthreshold slop factor, *V_T_* is thermal voltage (kT/q), Vth is transistor threshold voltage and *V_DD_* is the power supply voltage. The source current is mirrored to Delay_Cell_P and Delay_Cell_N for *I_REFN_* and *I_REFP_* respectively. According to Equation (6) and Equation (7), *I_REFP_* and *I_REFN_* should be of equal magnitude. Current mismatch between *I_REFP_* and *I_REFN_* will deteriorate the temperature linearity. To maintain the same magnitude of *I_REFP_* and *I_REFN_*, the optimal matching among current mirrors (M_1_/M_2_/M_6_ and M_4_/M_5_) is required. Thus, the maximum length of 20 µm is adopted for the current-mirror MOSFETs. The current generator is of simple structure but suffers from the influence of the power supply. Thus, a stable and clean power supply is required.

### 4.3. Quasi-Static D-Flip Flop

**Figure 7:**
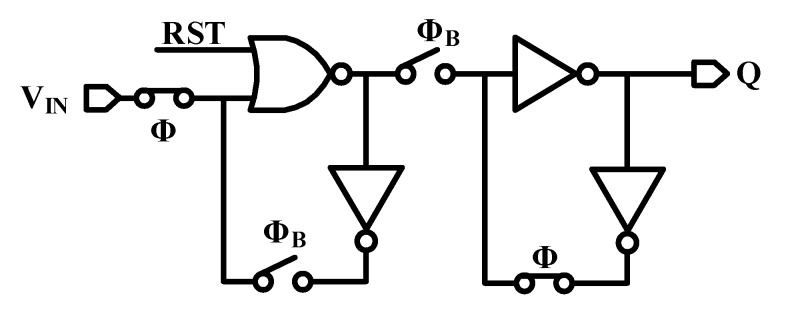
Quasi-static D-Flip Flop.

The counters consist of two cascaded dividers (divide by two) which are composed of quasi-static D-Flip Flop for low power consumption (shown in Figure 7). Note that no risk presents in the glitches. The D-Flip Flop adopts dual-feedback loop to remove the impact from leakage. When the counting value of Counter1 reaches *M*, the input clock is blocked, and all dividers in Counter1 and Counter2 stop working and hold the states. Counting value *P* in Counter2 is output as *Tcode* for measurement.

## 5. Simulation Results

**Figure 8:**
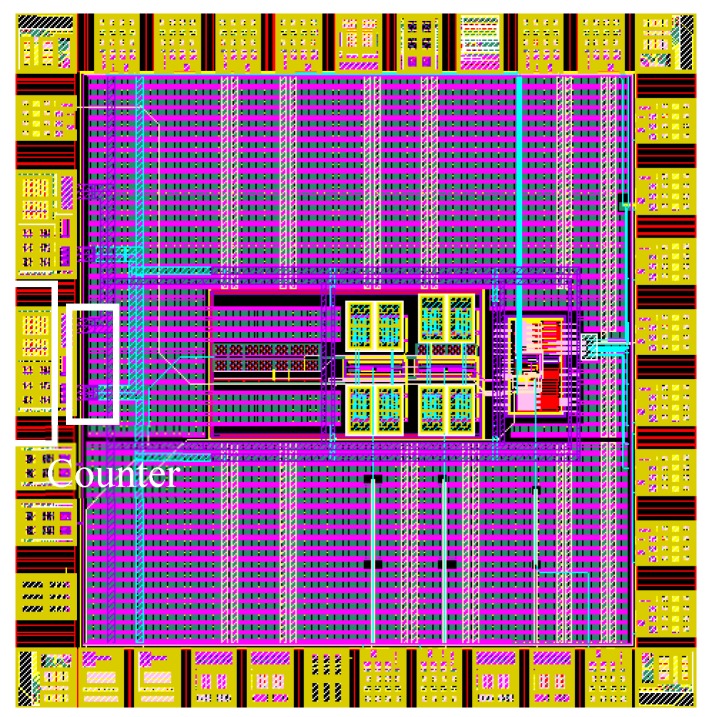
Layout.

**Figure 9:**
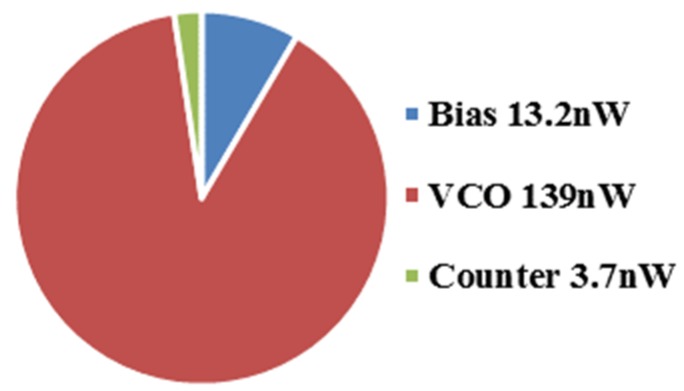
Power breakdown.

The proposed temperature sensor was designed in 130 nm CMOS process. It does not need external voltage, current and frequency reference. The layout is shown in Figure 8 and the active area is 0.065 mm^2^. The power supply voltage is 1 V to achieve low power consumption while keeping the bias current generator working properly under all corners and temperatures. The bias current was set to 6 nA with oscillation periods about 240 μs under typical corner and room temperature. The average power is 156 nW at 27 °C while VCO consumes 89%, as shown in Figure 9.

### 5.1. VCO Simulation

**Figure 10:**
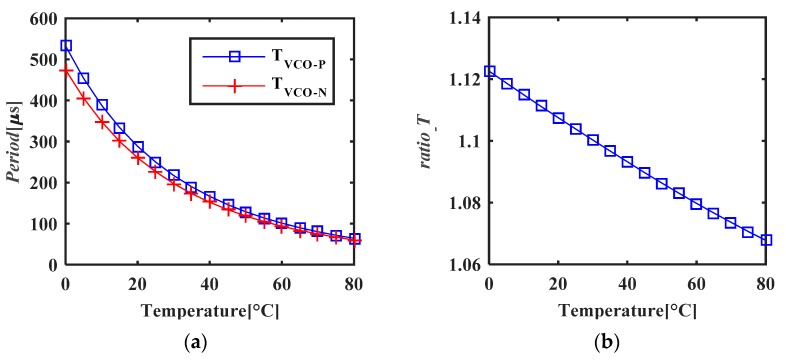
Simulated temperature performance of VCO (**a**) periods vs. temperature (**b**) *ratio_T* vs. temperature.

The proposed VCOs were designed and simulated. Figure 10 shows the simulated results. Since the bias generator works in sub-threshold region, the bias current has exponential relation to temperature. Accordingly, both VCO periods have exponential relation to temperature as shown in Figure 10a. Because of the threshold voltage difference between PMOS and NMOS, *T_VCO_P_* is larger than *T_VCO_N_*. *ratio_T* is shown in Figure 10b. It is inversely proportional to the temperature with an outstanding linearity which is in good accordance with the analysis in Equation (7).

### 5.2. Temperature Sensor Simulation

**Figure 11:**
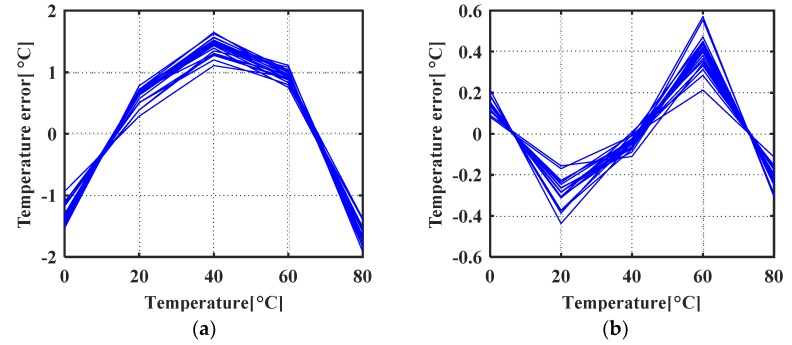
Simulated temperature error (**a**) after 1st order polyfit, (**b**) after 2nd order polyfit.

As shown in Figure 10b, *ratio_T* changes slightly over the temperature range of 0 °C to 80 °C. To maintain a 10-bit level resolution, Counter1 is designed to be 14 bits. According to the Monte Carlo simulations, *ratio_T* is always greater than 1 but less than 2, so Counter2 is set to be 15 bits. The proposed temperature sensor is simulated from 0 °C to 80 °C. Both MOSFET mismatches and MIM capacitor mismatch are taken into account and verified with 20 Monte Carlo runs. The standard deviation of the transistors’ mismatch and MIM capacitors’ mismatch are 0.3% and 0.03%, respectively, which are provided by the foundry. Figure 11a shows a temperature error of +1.65 °C /−1.84 °C after 1st order polyfit. Figure 11b shows a temperature error of +0.57 °C /−0.44 °C after 2nd order polyfit.

**Figure 12:**
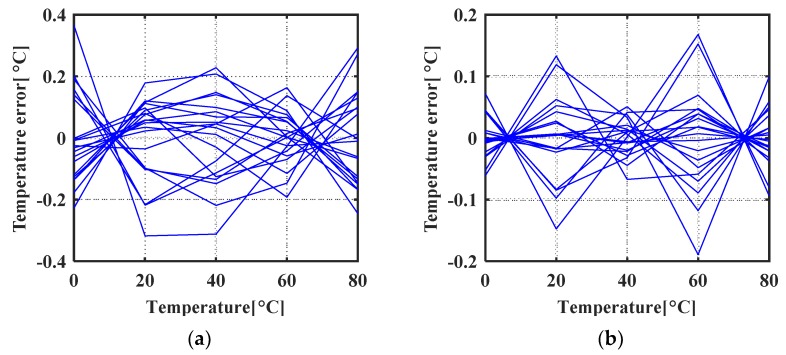
Simulated temperature error (**a**) after 1st order polyfit and systematic nonlinearity removal (**b**) after 2nd order polyfit and systematic nonlinearity removal.

The proposed temperature sensor exhibits a systematic error profile dominated by the nonlinearity of *ratio_T* across the temperature range which is in good accordance with the analysis in Equation (7). After 1st order polyfit and the nonlinearity removal (Figure 12a), a maximum temperature error of +0.37 °C/−0.32 °C was observed across a 0 °C to 80 °C temperature range. After 2nd order polyfit and the nonlinearity removal (Figure 12b), a maximum temperature error of +0.17 °C/−0.19 °C was observed.

**Figure 13:**
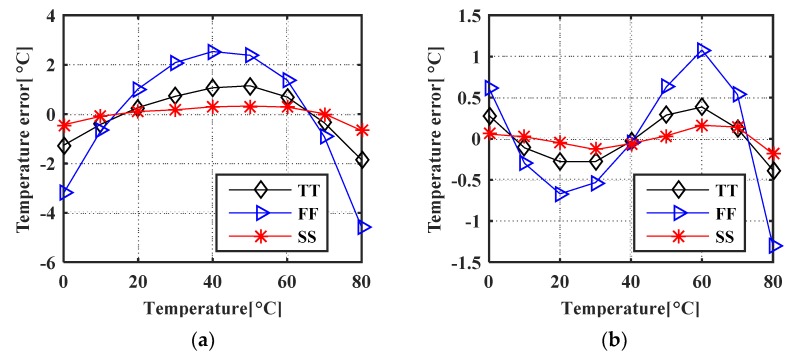
Simulated temperature error vs corners (**a**) after 1st order polyfit; (**b**) after 2nd order polyfit.

The proposed temperature sensor was verified under corners. The results are shown in Figure 13. The SS corner shows the best performance while the worst performance can be observed in the FF (Fast-Fast) corner. This is because in the FF corner, the MOSFETs have the lowest threshold voltage. When we increase the reference current, the oscillation period reduces. That results in a worse approximation in Equation (2) and poor linearity.

Table 1 compares the proposed sensor with the state-of-the-art temperature sensors. The proposed sensor does not need any external clock references or voltage regulators. It is designed with simple current mirrors and CMOS thyristors, and it achieves a competitive inaccuracy of +0.37/−0.32 °C and low power consumption of 156 nW within a compact area of 0.06 mm^2^.

## 6. Conclusions

A CMOS thyristor based temperature sensor was proposed in this paper. Two VCOs composed of CMOS thyristor with different threshold voltage were exploited. The period ratio between two VCOs extracts the temperature information. The ratio calculation is simply realized by two counters where a constant-value counter stops another free running counter. Therefore, the external clock reference was avoided. A diode-connected bias current generator was exploited for its simplicity and little impact on the temperature extraction. The prototype was designed in 130 nm CMOS process and occupies an active area of 0.06 mm^2^. According to the post-layout simulation, it achieves an inaccuracy of +0.37/−0.32 °C from 0 °C to 80 °C after 1st order polyfit and nonlinearity removal with a power consumption of 156 nW.

## Figures and Tables

**Table 1:** Summarizes the performance of the proposed sensor.

	[13]	[12]	[11]	[21]	This Work *
Technology [nm]	65	180	180	65	130
Area [mm^2^]	0.004	0.09	0.22	0.06	0.06
Supply Voltage [V]	0.85–1.05	1.2	1.2	1	1
External Clock	NO	NO	NO	YES	NO
Temperature Range [°C]	0–100	0–100	−20–80	0–100	0 to 80
Resolution [°C]	0.3	0.3	0.09	0.61	0.09
Conversion Time [s]	22 × 10^−6^	30 × 10^−3^	8 × 10^−3^	10 × 10^−6^–1	3.9
Power [nW]	154,000	71	570	488.3–0.17	156
Calibration	2-point	2-point	2-point	1-point	2-point
Inaccuracy [°C]	±0.9	+1.5/−1.4	±0.76	+1.5/−1.1	+0.37/−0.32

* Post layout simulation.
